# Urinary sulfated glycosaminoglycan insufficiency and chondroitin sulfate supplement in urolithiasis

**DOI:** 10.1371/journal.pone.0213180

**Published:** 2019-03-07

**Authors:** Thasinas Dissayabutra, Nuttiya Kalpongnukul, Kanokporn Chindaphan, Monpicha Srisa-art, Wattanachai Ungjaroenwathana, Maroot Kaewwongse, Kroonpong Iampenkhae, Piyaratana Tosukhowong

**Affiliations:** 1 STAR Unit of Renal Biochemistry and Stone Disease, Department of Biochemistry, Faculty of Medicine, Chulalongkorn University, Bangkok, Thailand; 2 Department of Chemistry, Faculty of Science, Chulalongkorn University, Bangkok, Thailand; 3 Division of Urology, Department of Surgery, Sunpasitthiprasong Hospital, Ubon Ratchathani Province, Thailand; 4 Division of Physiology, School of Medical Science, University of Phayao, Phayao, Thailand; 5 Renal Immunology and Transplant Research Unit, Faculty of Medicine, Chulalongkorn University, Bangkok, Thailand; University of Mississippi Medical Center, UNITED STATES

## Abstract

Familial members of urolithiasis have high risk for stone development. We observed the low sulfated glycosaminoglycan (GAG) excretion in urolithiasis patients and their descendants. In this study, we investigated urinary excretion of sulfated GAG, chondroitin sulfate (CS), heparan sulfate (HS) and hyaluronic acid (HA) in urolithiasis and their children, and explored the effect of CS and HA supplement in urolithic hyperoxaluric rats. The 24-hour urines were collected from urolithiasis patients (28) and their children (40), as well as healthy controls (45) and their children (33) to measure urinary sulfated GAG, CS, HS and HA excretion rate. Our result showed that urinary sulfated GAG and CS were diminished in both urolithiasis patients and their children, while decreased HS and increased HA were observed only in urolithiasis patients. Percentage of HS per sulfated GAG increased in both urolithiasis patients and their children. In hyperoxaluric rats induced by ethylene glycol and vitamin D, we found that CS supplement could prevent stone formation, while HA supplement had no effect on stone formation. Our study revealed that decreased urinary GAG and CS excretion are common in familial members of urolithiasis patients, and CS supplement might be beneficial in calcium oxalate urolithiasis prophylaxis for hyperoxaluric patients.

## Introduction

Familial members of urolithiasis have a greater risk of stone development than normal population [1]. Moreover, urolithiasis patients with positive family history of stone tended to have poor clinical outcomes [2, 3]. Our previous study demonstrated abnormal urinary excretion of phosphate, citrate and protein in urolithiasis patients and their children that had significant correlation with urinary supersaturation [4]. Hence, we proposed that increased risk of stone development in urolithiasis familial members is most likely caused by certain inherited metabolic disorders, which could be prevented by some appropriate management to correct these anomalies.

Previous studies demonstrated the function of urinary glycosaminoglycans (GAG) in stone formation. In short, chondroitin sulfate (CS) and heparan sulfate (HS) exerts a stone inhibitory effect [5, 6], while hyaluronic acid (HA) promotes crystal growth and retention [7]. For example, Momohara C. (2009) proposed that the inhibitory effect of urinary GAG and other macromolecules protected stone formation and renal cell injury [8]. Theoretically, urinary GAG was believed to have minimal effect on lithogenesis since their urinary concentrations were low [9]. However, due to the fact that deficiency of urinary GAG excretion is commonly found in stone formers [10–12], we hypothesized that GAG have more of a role in lithogenesis than previously recognized. Hence, we proposed that abnormal urinary GAG excretion might be inherited in familial members of patients with kidney stone disease, and supplementation of GAG might be useful in the prevention of stone formation in some high risk individuals. Objectives of this study aimed to demonstrated the abnormalities of urinary GAGs excretion in urolithiasis patients and their children who were considered to be at-risk, and investigate the effect of CS and HA supplement on stone formation in urolithic hyperoxaluric rats.

## Materials and methods

### Study design

A cross-sectional study was conducted from July 2013 to June 2015. Urolithiasis participants were patients who underwent stone removal surgery at the Urologic Division, Department of Surgery, Sunpasitthiprasong Hospital, Ubon Ratchathani Province, Thailand, radiographic study revealed no remnant of stone left after one month of surgery and committed to enrolled into the study.

Control subjects were randomly recruited from age- and gender-matched healthy individuals with negative history of kidney stones who resided in the communities within the distance of 30 kilometers from the hospital. The recruitment was done with the assistance of local village health volunteers, using community announcement for voluntary participation.

Children of urolithiasis patients and children of healthy control were the children of corresponding participants, and recruited with approval from their parents. The criteria for healthy control were subjects who had no pre-existing major organ dysfunction, such as brain, cardiac, liver and kidney, or having history of kidney stone disease. Subjects taking drugs that altered urinary compositions, for instance; diuretics, uricosuric drugs, calcium containing agents, etc. would be excluded (S1 Fig).

In addition, the exclusion criteria for incompleteness of urine collection were applied to all subjects. Participants who were suspected of incomplete 24-hour urine collection, and positive blood test were eliminated from the study. Regarding this, 146 subjects consisting of including 28 urolithiasis patients (U), 40 healthy control (C), 45 children of urolithiasis (UC), and 33 children of healthy control (CC) were enrolled.

### The 24-hour urine collection

Subjects were asked to collect their urine in a disposable sterile plastic vehicle with thymol preservation. Urine was collected for a whole 24 hours. The volume was measured, and a urine strip test (Analyticon® Biotechnologies AG, Germany) was done within 2 hours. Then, urine was incubated in ice during transport and stored in a -80°C refrigerator.

Exclusion criteria for urine were used to discard any urine suspected for incomplete 24-hour collection including; urine with total volume less of than 0.5 ml/kg/hour (12 ml/kg/day), or urinary creatinine excretion less than 27.6 mg/kg/day [13], and urine sample with positive blood test detected by urine strip.

### Urinary glycosaminoglycan detection in human urine

Total sulfated GAGs was measured by dye binding assay [14]. Capillary electrophoresis (CE) was used to measure urinary CS, HS and HA. In brief, urine treated with chondroitin ABC overnight contained in 50 μm-diameter fused-silica capillary tube with 50 mM monosodium phosphate (NaH_2_PO_4_) and butylamine buffer under -15 kV reversed-polar mode was measured at 195 nm light absorption. [15] Chondroitin sulfate sodium salt (Sigma-Aldrich) heparan sulfate sodium salt and sodium hyaluronate (Cockscomb, TCI Europe N.V) were used as standards.

### Urolithic hyperoxaluric rats

Rats were chosen for the present study because of rats produced adequate urine for our investigation. Various species of rats were generally used in urolithiasis experiments [16], and male was more preferable than female animals which was resistant to develop stone in hyperoxaluric condition [17]. In the present study, 8-week-old male Wistar rats were purchased from Nomura International Siam (Thailand), and had been tested for stone inductility using ethylene glycol protocol [18]. All rats were housed under pathogen-free, 12-hour light/dark cycle condition with 21 to 25°C temperature and 40–60% relative humidity and fed by regular diet. They received ad libitum access to food and water. Rats were randomly divided into 5 groups; Control, Urolithiasis, Urolithiasis with CS supplement (CS supplement), Urolithiasis with HA supplement (HA supplement) and Urolithiasis with simultaneous CS and HA supplement (CS&HA supplement). Each group comprised of 9 rats. All hyperoxaluric rats were induced by ethylene glycol 500 mg/kg intraperitoneal injection once (day 1), and vitamin D 0.1 μg intraperitoneal injection for 5-consecutive day (day 1 to 5) [17], while control rats were injected with normal saline. CS (Hangzhou Hyper Chemicals Limited, China) was supplemented for 40 mg/kg/day and HA (Hangzhou Hyper Chemicals Limited, China) for 10 mg/kg/day in the corresponding groups by gavage. Body weight, food and water intake were recorded, and 24-hour urine was collected on day 0, 8 and 15 in metabolic chamber to evaluate urine volume, creatinine, CS and HA measurement. Tail blood was drawn on day 0, 8 and 15 to measure serum creatinine and alanine aminotransferase (ALT) as indicators of kidney and liver functions. Rats were sacrificed at the end of day 15, then kidneys were harvested and preserved in 10% formalin for further study. Procedures were performed under inhalation anesthesia (carbon dioxide).

Urine creatinine was measured by electrochemiluminescent technique using COBAS C6000 (Roche). CS and HA were measured by ELISA method (MyBioSource, USA).

Kidneys were bisected and totally submitted for formalin-fixed paraffin-embedded (FFPE) tissue processing. Then they were cut into 4 μm section, stained by H&E. Microscopic examination was performed to evaluate renal tissue. Calcium oxalate crystal was identified by positive birefringent under polarized microscope. Urolithiasis grading was scored by number of nephron with calcium oxalate deposition in tubules: Grade 0 referred to no calcium oxalate deposit in any nephron; Grade 1 contained less than 5 nephrons with calcium oxalate deposition per section; Grade 2 contained 5–19 nephrons with calcium oxalate deposition per section; Grade 3 contained 20–50 nephrons with calcium oxalate deposition per section and Grade 4 contained more than 50 nephrons with calcium oxalate deposition per section.

### Statistical analysis

Continuous variables were expressed as mean ± standard deviation and categorical variables as median and interquartile range. All data were analyzed by SPSS v 22.0 (IBM, USA). Valuables were analyzed with a student t-test or ANOVA and Bonferroni post-hoc analysis for data greater than two groups. Man-Whitney U test was used to analyze the incidence of urolithiasis and degree of urolithiasis. Statistical significance was determined as p-value < 0.05.

### Ethical consideration

The study in human samples was approved by the Ethics Committee for Research in Human Subjects in the Fields of Thai Traditional and Alternative Medicine, and by the Ethics Committee of Sunpasitthiprasong Hospital. Written consent or assent was obtained from all participants after a detailed explanation of the procedures was provided. In addition, parental approval was obtained at the same time for all descendant subjects. The animal study was approved by the Laboratory Animal Research Center–University of Phayao (LARCUP) and Chulalongkorn University Animal Care and Use Committee (CU-ACUC).

## Results and discussion

### Urinary GAG excretion in urolithiasis patients and their children

There was no difference in gender, age and body mass index between each group (Table 1). Among urolithiasis, most of stones removed from urolithiasis patients (93%) were identified by Fourier-transform infrared spectroscopy (FTIR) to comprise of calcium, while only 7% contained uric acid as the main composition. Urinary excretion of sulfated GAG and CS were lowered in urolithiasis patients and their children compared with corresponding control groups (Table 2). HS excretion was decreased and HA was elevated exclusively in urolithiasis patients. Furthermore, the percentage of HS per total sulfated GAG was increased in urolithiasis patients and their children, while the percentage of CS was not different (Table 3). Urinary sulfated GAG excretion was positively correlated with urinary citrate and uric acid excretion (r = 0.390, p<0.001 and r = 0.179, p = 0.031, respectively), but negatively correlated with albumin excretion (r = -0.195, p = 0.018). There was no correlation obtained between urinary sulfated GAG and calcium, oxalate, magnesium, phosphate. sulfate, chloride and sodium excretion, as well as urinary pH and supersaturation level (data not shown).

**Table 1:** Baseline characteristics of healthy controls, urolithiasis patients, children of control (CC) and children of urolithiasis (UC).

		Control	Urolithiasis	p-value	CC	UC	p-value
	*N*	*40*	*28*		*33*	*45*	
**Gender (%male)**		52.50%	64.30%	0.171	45.50%	48.90%	0.764
**Age (years)**		44.6 + 9.2	46.1 + 9.8	0.52	20.2 + 7.5	19.1 + 7.9	0.547
**Body mass index (kg/m**^**2**^**)**		24.5 + 3.8	24.1 + 3.6	0.676	20.8 + 3.7	19.6 + 3.8	0.181

**Table 2:** Urinary glycosaminoglycan excretion of healthy controls, urolithiasis patients, children of control (CC) and children of urolithiasis (UC).

	Control	Urolithiasis	CC	UC
**Total sulfated GAGs (mg/day)**	46.2±40.0	12.5±11.8*	43.8±23.1	24.4±10.4^#^
**Chondroitin sulfate (mg/day)**	30.30±20.11	6.63±4.39*	24.77±17.87	14.51±8.35^#^
**Heparan sulfate (mg/day)**	6.94±5.53	3.67±3.60*	6.32±4.20	5.97±3.77
**Hyaluronic acid (mg/day)**	8.65±5.60	15.67±11.51*	3.11±2.56	4.41±4.52

*p < 0.05 compared with Control

^#^p < 0.05 compared with CC

**Table 3:** Chondroitin sulfate and heparan sulfate fraction in total sulfated glycosaminoglycan in urine of healthy controls, urolithiasis patients, children of control (CC) and children of urolithiasis (UC).

	Control	Urolithiasis	CC	UC
**Chondroitin sulfate/Total sGAGs (%)**	61.7±21.4	56.6±24.5	53.5±23.4	56.9±18.2
**Heparan sulfate/Total sGAGs (%)**	15.7±8.0	23.2±16.8*	15.0±8.5	22.8±7.6^#^

*p < 0.05 compared with Control

^#^p < 0.05 compared with CC

Chondroitin sulfate is the most abundant GAG detected in urine in normal population, followed by hyaluronic acid, heparan sulfate, dermatan sulfate and keratan sulfate, respectively [19]. Many researchers reported low sulfated GAG excretion in urolithiasis patients [20, 21]. Our study was the first to demonstrate that children of stone formers had decreased urinary sulfated GAG excretion similar to their parents, but with a lesser degree, asymptomatic urinary crystallization which expended GAG. Regarding excretion rate, we found that CS was diminished in urine of both patients and their children, while HS depletion was observed solely in urolithiasis patients. However, CS excretion appeared to be proportionally lower corresponded with total GAG reduction, but HS excretion was similarly increased in both stone formers and their children.

Recent evidence has been confirmed that CS and HS are stone inhibitors. The anti-lithogenic property of CS depends on the structure and sulfate groups. Some scientists also claimed that CS in children may contain higher anti-stone potency than CS found in adults [8, 22]. Regarding low urinary CS observed in children of urolithiasis, we believed that this might be one of the risks related to high probability of stone development in urolithiasis familial members.

In HS, strong anti-lithogenic activity was demonstrated [7, 9]. HS production from renal epithelial cells was upregulated when exposed to oxalate or calcium oxalate crystal [23, 24]. The increased proportion of HS per sulfated GAG may indicate tubular injury caused by oxalate, crystal or stone. Furthermore, the present study indicated an elevation of urinary HA in urolithiasis patients, corresponding with previous studies. HA is upregulated during tubular injury and inflammation, and promotes crystal growth and adhesion to tubular cells by binding with CD44 [9].

The causes of sulfated GAG deficiency in urine of urolithiasis patients could be due to insufficient GAG production or excessive GAG consumption during stone formation. However, the urinary sulfated GAG depletion in urine of children of urolithiasis patients without stone formation suggests that these susceptible children had inherited an abnormality that causes deficient GAG synthesis from their parents. Another possible explanation is that these children may form small urinary crystals that consume GAG but does not further develop into stone. This hypothesis coincided with the increased HS fraction but normal HA in total urinary GAG found in children of urolithiasis patients and was supported by our previous studies, performed in the same group of participants, which demonstrated that these children had high urinary supersaturation index similar to the stone-former patients [4].

### Stone formation in urolithic hyperoxaluric rats supplemented with CS and/or HA

During the experiment, each rat in urolithiasis and CS&HS supplement groups were succumbed. Regarding this, there were eight rats remaining in these two groups at the end of the study. Conclusively, we found that urolithiasis and hyperoxaluric rats supplemented with CS had lower body weight at day 15 when compared with control group (Table 4). Their body weights were not correlated with food and water intake, urine output, serum ALT, creatinine, and renal pathologies. Furthermore, there was no association between HA supplement and body weight in hyperoxaluric rats.

**Table 4:** Body weight, urine output, food and water intake of urolithic hyperoxaluric rats induced by ethylene glycol and vitamin D.

	Body weight (g)			Urine output (ml/day)			Food intake (g/day)			Water intake (ml/day)		
Group	Day 0	Day 8	Day 15	Day 0	Day 8	Day 15	Day 0	Day 8	Day 15	Day 0	Day 8	Day 15
Control	272.3±26.5	317.7±15.7	352.5±11.5	9.5±4.2	17.2±6.5	12.5±4.0	18.8±3.9	22.8±2.0	21.5±2.7	31.6±5.6	35.6±4.8	36.1±3.6
Stone	271.7±27.8	285.5±32.8	317.6±29.4*	11.1±5.4	23.9±12.2	19.7±13.8	18.4±6.0	16.9+±6.0	21.1±2.8	33.2±7.7	46.0±15.2	37.2±7.2
CS supplement	262.6±23.9	277.3±9.5	311.2±10.6*	14.1±5.6	29.3±8.7	23.7±5.4	18.6±5.9	18.2±6.2	21.0±3.4	35.5±8.0	50.0±6.7	50.5±12.0
HA supplement	265.8±32.3	302.4±22.6	332.6±13.7	12.7±7.8	20.4±7.8	25.0±15.6	16.5±6.6	21.2±3.6	20.8±3.5	33.0±14.2	44.6±15.2	37.9±10.3
CS&HA supplement	266.6±32.4	295.6±29.4	331.1±22.9	13.7±3.8	22.7±11.8	24.9±10.7	18.2±4.4	18.5±6.3	22.7±4.9	33.7±3.6	45.4±8.3	40.6±12.4

*p < 0.05 compared with day 0

At day 8 and day 15, urinary CS excretion was increased in rats supplemented with CS (CS and CS&HA supplement groups), while HA excretion was elevated in rats supplemented with HA (HA and CS&HA supplement) (Table 4).

**Fig 1:**
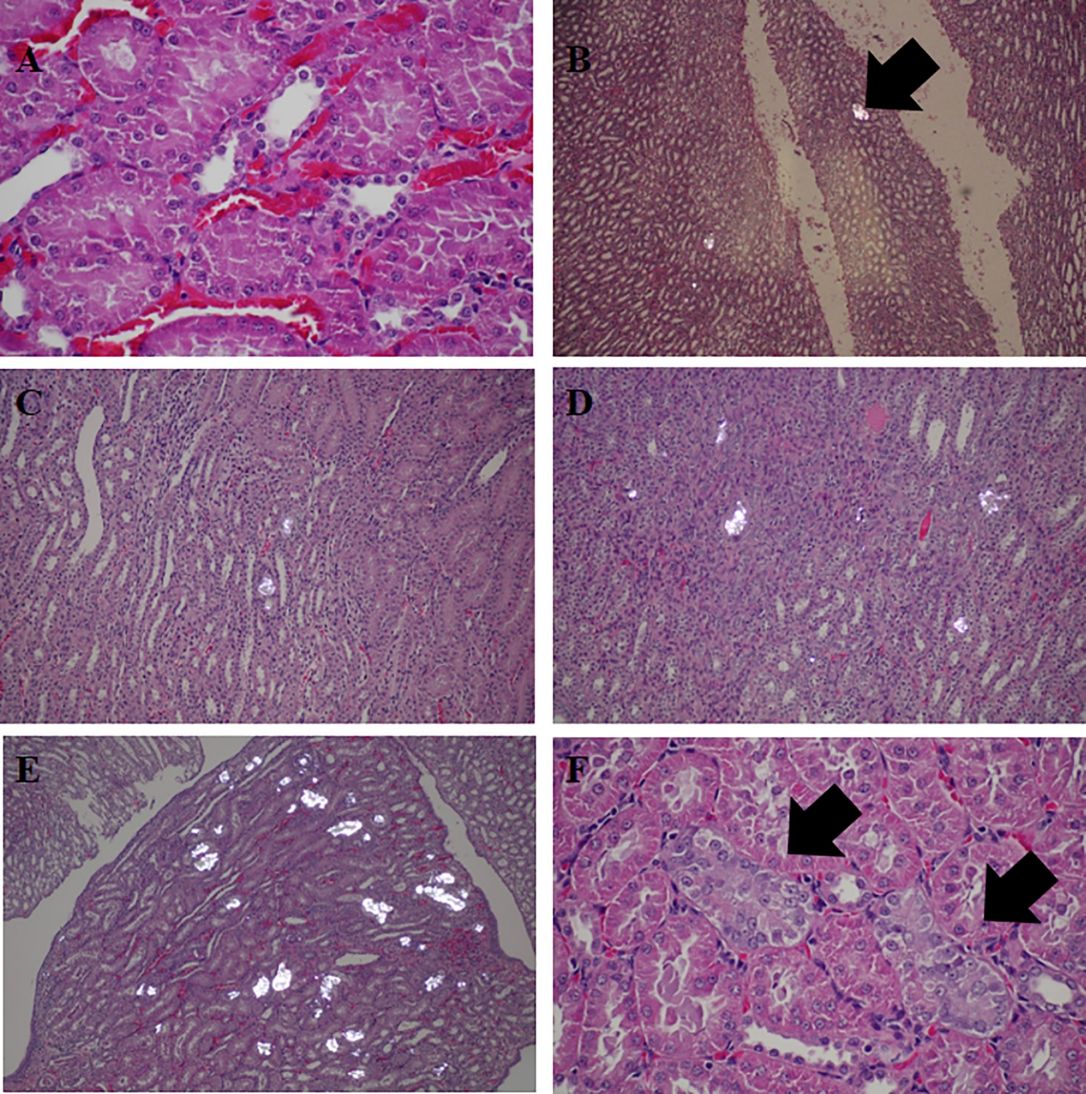
Urolithiasis grading in renal tissue with H&E staining visualized by light microscope (LM) and polarized microscope (PM). Grade 0 contained calcium oxalate crystal deposit in all section (A, LM x40); Grade 1 contained less than 5 nephrons with calcium oxalate crystal deposition in distal tubular lumen (B, PM, x20); Grade 2 contained 5–19 nephrons with calcium oxalate deposition (C, PM x20); Grade 3 contained 20–50 nephrons with calcium oxalate deposition mostly in distal tubular regions, and occasionally in proximal tubular regions (D, PM x20); Grade 4 contained more than 50 nephrons with calcium deposition in both distal and proximal tubular regions (E, PM x 20); Tubular injury characterized by thin brush border, mildly enlarged nuclei, coarse chromatin and visible nucleoli was detected in the association with urolithiasis formation (E, LM x40).

Histopathologic study of rat kidney revealed that kidney stones were detected in kidney tissue of all rats in urolithiasis group, while none of control rats developed stone (Tables 5 and 6). Approximately 33.3% kidneys of hyperoxaluric rats supplemented with CS developed stone, while 62.5% of CS&HA supplemented rats and all HA supplemented rats had stones. Degree of urolithiasis grading was the most severe in urolithiasis rats. Urolithiasis was ameliorated in hyperoxalurix rats supplemented with CS (both CS supplemented and CS&HA supplemented groups compared with urolithiasis group, p = 0.002 and p = 0.021, respectively). However, HA supplementation showed no effect on stone formation. None of the rat kidneys developed tubulo-interstitial fibrosis in the present study (Fig 1).

**Table 5:** Urinary chondroitin sulfate (CS) and hyaluronic acid (HA) excretion and incidence of urolithiasis in urolithic hyperoxaluric rats.

Group	Urine CS (mg/g creatinine)			Urine HA (mg/g creatinine)			Stone formation (cases/total)
	Day 0	Day 8	Day 15	Day 0	Day 8	Day 15	
**Control**	1.18+0.43	1.11+0.30	1.35+0.36	0.13+0.11	0.12+0.09	0.11+0.09	0/9
**Urolithiasis**	1.39+0.40	1.39+0.39	1.81+0.64	0.13+0.10	0.15+0.12	0.19+0.12	8/8
**CS supplement**	1.37+0.48	3.37+1.33*	4.68+0.68*	0.18+0.09	0.15+0.07	0.18+0.10	3/9^#^
**HA supplement**	1.28+0.37	1.77+0.63	1.88+0.59	0.18+0.10	0.84+0.29*	0.87+0.18*	9/9
**CS&HA supplement**	1.36+0.47	3.12+1.39*	4.39+0.57*	0.17+0.06	0.74+0.14*	1.01+0.38*	5/8^#^

*p < 0.05 compared with day 0

^#^p < 0.05 compared with Control

**Table 6:** Severity score of urolithiasis in urolithic hyperoxaluric rats.

Group	Urolithiasis grade		Tubulo-interstitial fibrosis
	Median	Range	Median
Control	0	0	0
Urolithiasis	3	2–4	0
CS supplement	0*	0–3	0
HA supplement	3	1–4	0
CS & HA supplement	1*	0–4	0

*p < 0.05 compared with Urolithiasis group

Our study demonstrated that CS supplement was effective in prevention of urolithiasis in most of hyperoxaluric rats, whereas HA supplement had no effect on urolithiasis formation, Combination of CS and HS supplementation yielded no advantage over single CS supplementation in stone prophylaxis in this study. Regarding our results, we proposed that CS supplement should be beneficial in the prevention of urolithiasis, while HA supplement appeared to have no effects in stone formation. The stone inhibitory effect of CS appeared to mitigate calcium oxalate crystal formation or crystal growth.

The limitation of this study was that we could not demonstrated the effect of CS and HA supplement on tubulo-interstitial fibrosis due to the short duration of experimentation. This time restriction was because of our observation in pilot study revealed the significantly increased mortality rate of hyperoxaluric rats after 15-days of experiment. Low nephrotoxic substance such as hydroxyl L-proline should be used instead of ethylene glycol to induce hyperoxaluria for validation of glycosaminoglycan effects on tubulo-interstitial fibrosis and other urolithiasis complications. Despite of the limitation, this was the first study to demonstrate urinary GAG insufficiency in children of urolithiasis and the benefit of CS supplement in calcium oxalate stone prevention in hyperoxaluric rats. Further investigation to verify the mechanism and effect of CS supplement on urolithiasis patients should be carried out.

## Conclusion

Children of urolithiasis patients had urinary sulfated GAG insufficiency but increased HS fraction in total sulfated GAG excretion, similar to urolithiasis patients. In addition, CS supplementation in urolithic hyperoxaluric rats can prevent stone formation, while HA supplementation had no effect on lithogenesis.

## Supporting information

S1 Fig Flow diagram to demonstrate the enrollment and exclusion of each group of subjects. (TIF)
