# Supplementary figures and images for: Urinary sulfated glycosaminoglycan insufficiency and chondroitin sulfate supplement in urolithiasis
Source: PLoS One. 2019 Mar 7;14(3):e0213180. doi: 10.1371/journal.pone.0213180 (PMC6405089; doi:10.1371/journal.pone.0213180)

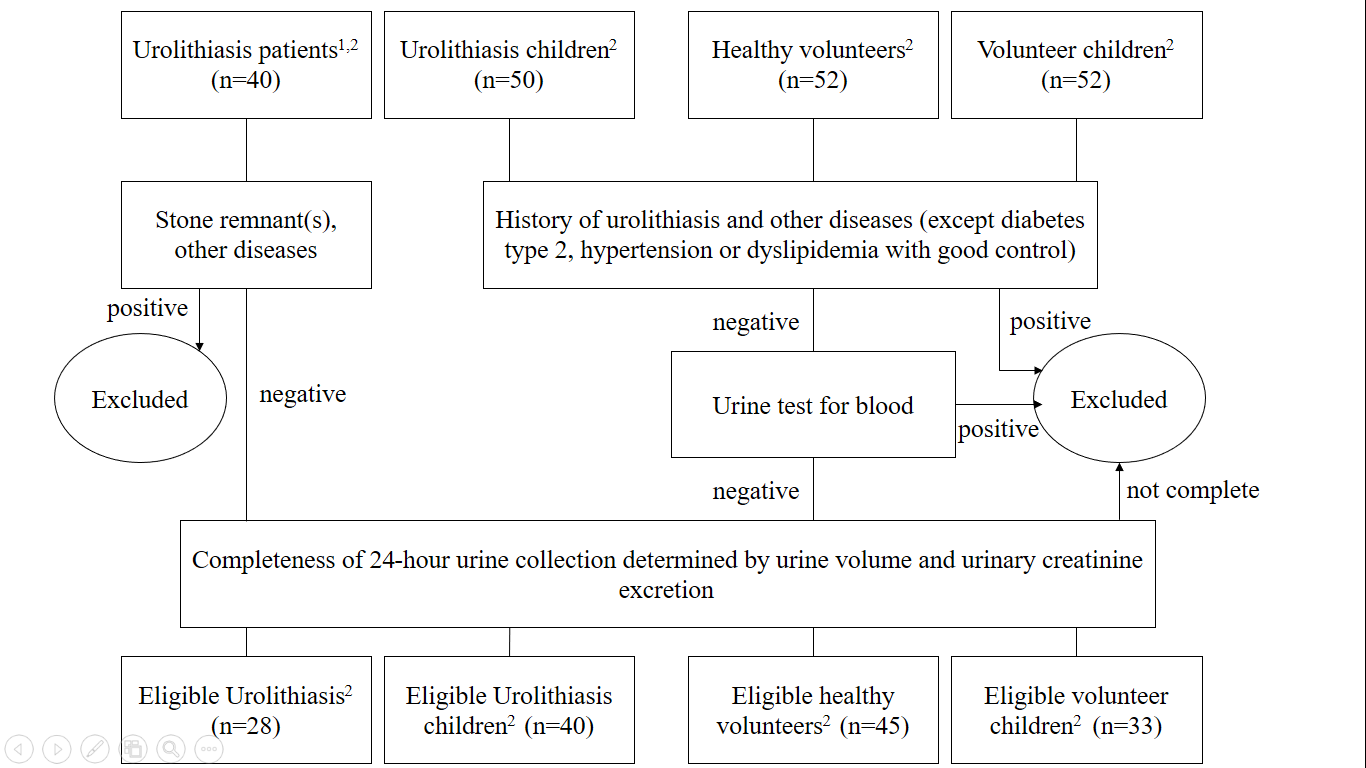

Supplement: S1 Fig — (TIF) [file pone.0213180.s001.tif]
